# Diagnostic performance of deep learning-based vaginal microecological morphology assessment for bacterial vaginosis and vulvovaginal candidiasis

**DOI:** 10.3389/fmicb.2026.1898300

**Published:** 2026-08-07

**Authors:** Peng Zhang, He-Mei Li, Hui-Hui Gao, Lin Yan

**Affiliations:** 1 Reproductive Medicine Center, The Central Hospital of Wuhan, Tongji Medical College, Huazhong University of Science and Technology, Wuhan, China; 2 Department of Obstetrics and Gynecology, The Central Hospital of Wuhan, Tongji Medical College, Huazhong University of Science and Technology, Wuhan, China; 3 Key Laboratory for Molecular Diagnosis of Hubei Province, The Central Hospital of Wuhan, Tongji Medical College, Huazhong University of Science and Technology, Wuhan, China

**Keywords:** automated morphology assessment, bacterial vaginosis, deep learning, vaginal microecology, vulvovaginal candidiasis, diagnostic accuracy

## Abstract

**Background:**

Routine wet mount microscopy for bacterial vaginosis (BV) and vulvovaginal candidiasis (VVC) is rapid but operator-dependent and lacks ecological context. We evaluated a deep learning–based vaginal microecological morphology assessment (DL-VMM) system integrated in the GE6000 automated vaginal secretion analyzer.

**Methods:**

In a prospective cross-sectional study, 500 symptomatic women provided vaginal smears. DL-VMM was compared with routine wet mount microscopy against a composite reference standard (Nugent score, Amsel criteria, fungal microscopy). Key morphological features including clue cells, blastospores, hyphae, trichomonads, leukocytes, epithelial cells, and cleanliness grade were assessed. Diagnostic accuracy and Cohen’s kappa were calculated. We also analyzed discordant cases to explore sources of disagreement.

**Results:**

For BV diagnosed by clue cells, DL-VMM achieved 100.00% sensitivity (95% CI: 94.19–100.00%), 98.86% specificity (97.37–99.58%), and κ = 0.95. For VVC by blastospores, sensitivity was 99.32% (95.85–99.96%), specificity 98.87% (96.94–99.70%), κ = 0.97; by hyphae, sensitivity 100.00% (97.68–100.00%), specificity 98.81% (97.04–99.63%), κ = 0.98. Total agreement between DL-VMM and manual microscopy for all parameters ranged from 96.40% to 99.60% (κ 0.89–0.98). No statistically significant differences were found between methods (all *P* > 0.05).

**Conclusion:**

This prospective diagnostic study demonstrates that the DL-VMM system achieves high diagnostic accuracy for BV and VVC, with substantial to almost perfect agreement with routine wet mount microscopy. The automated system eliminates operator dependence, provides rapid results, and may standardize vaginitis diagnosis, although discordances, though minor, warrant awareness and potential manual review in equivocal cases.

## 1 Introduction

Vaginitis represents one of the most prevalent gynecological disorders encountered in clinical practice worldwide, imposing a substantial clinical and public health burden on reproductive-aged women (Amsel et al., 1983). Bacterial vaginosis (BV) and vulvovaginal candidiasis (VVC) are the leading etiologies of symptomatic vaginitis, manifesting clinically with abnormal vaginal discharge, pruritus, malodor, and local irritation (Sobel, 1997). Both conditions arise from disruptions of the vaginal microbiota homeostasis and are associated with elevated risks of recurrent infection, adverse obstetric outcomes, pelvic inflammatory disease, and increased susceptibility to sexually transmitted infections (Bradshaw et al., 2025; Hillier et al., 1993; Sobel, 2007).

BV is characterized by the depletion of hydrogen peroxide-producing lactobacilli and the overgrowth of anaerobic bacteria, most notably *Gardnerella vaginalis*, accompanied by elevated vaginal pH and the presence of clue cells (Chen et al., 2025; Nugent et al., 1991; Ravel et al., 2011). In contrast, VVC is predominantly caused by *Candida* species, with *Candida albicans* accounting for the majority of cases, and its pathogenesis is modulated by host immune status, hormonal fluctuations, and microbial ecological imbalance (Buchta et al., 2025; Nyirjesy, 2008; Sutton et al., 2026). Accurate and timely diagnosis of BV and VVC is critical for targeted antimicrobial therapy, reducing recurrence, and preventing long-term complications (Workowski et al., 2021).

Routine wet mount microscopy, including saline wet mount and 10% potassium hydroxide (KOH) preparation, remains the first-line diagnostic approach in most clinical settings due to its rapidity, low cost, and wide availability (Muzny and Kardas, 2020). This conventional method enables direct visualization of clue cells, fungal blastospores and hyphae, *Trichomonas vaginalis*, leukocytes, and assessment of vaginal cleanliness grade (Hemalatha et al., 2013). However, wet mount microscopy has well-documented limitations: diagnostic performance is highly operator-dependent, with substantial inter-observer variability (Kenyon et al., 2013); sensitivity declines significantly in cases with low pathogen load or subtle morphological features (Donders, 2007); and it provides limited quantitative and ecological insights into the overall vaginal microenvironment (Chen et al., 2025). These drawbacks compromise diagnostic consistency, especially in high-volume laboratories or resource-limited settings.

In recent years, deep learning-based digital image analysis has emerged as a transformative tool in clinical laboratory diagnostics, offering objective, automated, and standardized evaluation of cytological and histological specimens (Cauci, 2004). The deep learning-based vaginal microecological morphology assessment (DL-VMM) system integrated into the GE6000 automated vaginal secretion analyzer utilizes convolutional neural network (CNN) architecture with spatial attention mechanisms to achieve intelligent recognition, segmentation, and classification of key morphological indicators in vaginal smears (Donders et al., 2009; Ravel et al., 2011). This automated platform is designed to overcome the limitations of manual microscopy by providing quantitative microecological evaluation, including Nugent-compatible scoring, lactobacilli grading, and pathogen detection (Chen et al., 2025; Ravel et al., 2011).

To date, prospective diagnostic studies directly comparing the performance of DL-VMM with routine wet mount microscopy for BV and VVC in a large clinical cohort remain limited. Therefore, we conducted this prospective, cross-sectional diagnostic study to evaluate the diagnostic accuracy, agreement, and reliability of the GE6000-integrated DL-VMM system relative to conventional wet mount microscopy, using a composite reference standard for BV and VVC. We hypothesized that the automated DL-VMM system would exhibit non-inferior or superior diagnostic performance, with excellent concordance to manual microscopy, thereby supporting standardized and objective vaginitis diagnosis in routine clinical practice.

## 2 Materials and methods

### 2.1 Study design

This was a prospective, cross-sectional diagnostic study designed to compare the performance of a deep learning-based vaginal microecological morphology assessment system (DL-VMM) with that of routine wet mount microscopy for diagnosing bacterial vaginosis (BV) and vulvovaginal candidiasis (VVC). The study was conducted at the Department of Obstetrics and Gynecology, The Central Hospital of Wuhan, affiliated with Tongji Medical College, Huazhong University of Science and Technology. All microscopists and reference standard adjudicators were blinded to the DL-VMM results.

### 2.2 Study population

Women of reproductive age (18–45 years) presenting with vaginal symptoms (e.g., abnormal discharge, itching, malodor, or irritation) were consecutively recruited from outpatient gynecology clinics.

*Inclusion criteria:* participants with clinical suspicion of vaginitis who provided informed consent.

*Exclusion criteria:* use of antibiotics, antifungals, or probiotics within 4 weeks prior to sampling; menstruation at the time of sampling; pregnancy; or known immunosuppressive disorders.

Demographic and clinical data, including age, symptom duration, medical history, and prior treatment, were collected at enrollment. Based on a composite reference standard (see section 2.7), participants were categorized into BV, VVC, mixed infection (BV + VVC), or normal flora groups. All eligible patients were consecutively enrolled until the target sample size of 500 was reached; no other selection criteria were applied.

### 2.3 Sample collection and preparation

Two vaginal swabs were collected from the lateral vaginal wall and posterior fornix under aseptic conditions:

① *Wet mount microscopy sample:* One swab was immediately used to prepare a saline wet mount and a 10% potassium hydroxide (KOH) preparation for conventional microscopic examination to identify clue cells, epithelial cells, leukocytes, *Trichomonas vaginalis*, and fungal elements (blastospores and hyphae).
② *DL-VMM sample*: The second swab was used to prepare a thin Gram-stained or methylene blue-stained smear. The slide was digitized using a high-resolution digital microscope equipped with a CMOS camera (40 × objective; resolution ≥ 0.25 μm/pixel) integrated into the GE6000 system.

### 2.4 Materials and reagents

The GE6000 automatic vaginal secretion analyzer (deep learning–based vaginal microecological morphology assessment system, DL-VMM) and its consumables were obtained from Hunan Shengtong Technology Co., Ltd. (Hunan, China). Gram staining solution (catalog no. 77730) was purchased from Millipore (Burlington, MA, United States). An Olympus CX41 microscope was obtained from Leica Microsystems GmbH (Wetzlar, Germany).

### 2.5 Routine wet mount microscopy

Conventional saline and 10% KOH preparations were performed according to standard procedures (Amsel et al., 1983; Nugent et al., 1991). Gram-stained smears were examined following the *National Clinical Laboratory Operating Procedures* (Fourth Edition). Evaluated parameters included epithelial cells, leukocytes, blastospores, hyphae, *Trichomonas vaginalis*, clue cells, and vaginal cleanliness grade. Clue cells were defined as epithelial cells with adherent bacteria obscuring cell borders. Detection of *Candida* hyphae, pseudohyphae, or blastospores supported the diagnosis of vulvovaginal candidiasis (VVC). Blastospores, hyphae, *T. vaginalis*, and clue cells were considered positive if present. Cleanliness grades I-II were classified as normal (negative), and grades III–IV as inflammation (positive) (Table 1). All readings were performed by two experienced microscopists; discrepancies were resolved by consensus. Inter-observer variability between the two microscopists was not formally quantified, but all final readings were consensus-based.

**TABLE 1:** Criteria for vaginal smear cleanliness grading (Ison and Hay, 2002; Shang et al., 2015).

Cleanliness grade	Bacilli	Cocci	Epithelial cells	Leukocytes (or pus cells) (cells/HP)
I	Many	–	Full field	0–5
II	Moderate	Few	1/2 field	5–15
III	Few	Many	Small amount	15–30
IV	–	Large number	–	> 30

### 2.6 GE6000 automated vaginal secretary analyzer integrated with the DL-VMM system

The DL-VMM system operates through three sequential main processes. First, data preprocessing and augmentation involved standardizing digital smear images via resolution alignment, color normalization, and illumination balancing. Background noise such as mucus and debris was removed using morphological filters. Data augmentation techniques including rotation, brightness adjustment, flipping, Mixup, Cutout, and overlap simulation were applied to enhance model robustness. Second, model development and training employed a convolutional neural network (CNN) based on the ConvNeXt Base architecture integrated with a spatial attention mechanism. A multi task joint learning strategy was used for simultaneous detection, segmentation, and classification of key morphological features, including clue cells, blastospores, hyphae, lactobacilli density, and epithelial cells. Transfer learning was applied using pretrained weights from biomedical image datasets. Training was conducted on 80% of the annotated dataset, approximately 12,000 cells, with validation on the remaining 20%. The optimizer was AdamW with a learning rate of 1 × 10^−4^ and weight decay of 0.05. Training proceeded for 200 epochs with early stopping, performed on NVIDIA A100 GPUs with 40 GB memory and mixed FP16 precision. Third, output classification and reporting generated a comprehensive diagnostic report for each sample. This report included vaginal microecological morphology assessment, epithelial morphology evaluation, white blood cell count with inflammation score, lactobacilli grading, clue cell presence and score, Nugent score with quantitative dysbiosis index ranging from 0 to 1, and detection of fungal elements for VVC prediction. The training dataset was derived from the 500 enrolled patients (each providing multiple annotated cells), resulting in approximately 12,000 annotated cells; no additional external cases were used for training.

### 2.7 Reference standard

A composite reference standard was used to define true diagnostic outcomes:

① BV: Nugent score ≥ 7 or ≥ 3 positive Amsel criteria (discharge, pH > 4.5, clue cells, fishy odor).
② VVC: compatible symptoms plus visible yeast elements (blastospores or hyphae) on microscopy.
③ Mixed vaginitis: simultaneous BV and VVC criteria met.
④ Normal flora: Nugent ≤ 3 with no fungal or *Trichomonas* elements.

All reference diagnoses were adjudicated by two senior microbiologists blinded to DL-VMM predictions.

### 2.8 Statistical analysis

Quantitative variables were expressed as mean ± SD or median (IQR); categorical variables as counts (%). Sensitivity, specificity, positive predictive value, negative predictive value, accuracy, and area under the curve (AUC) were calculated. Agreement between DL-VMM and manual microscopy for all morphological parameters was assessed using Cohen’s kappa (κ). McNemar’s test (or chi-square test where applicable) compared paired proportions. All tests were two-sided, with *P* < 0.05 considered statistically significant. SPSS v27.0 and MedCalc v22.2 were used for analyses. For discordant cases, we tabulated false-positive and false-negative counts for each parameter and examined their distribution across disease categories and morphological features.

### 2.9 Ethical statement

The study protocol was reviewed and approved by the Ethics Committee of The Central Hospital of Wuhan, Tongji Medical College, Huazhong University of Science and Technology (Approval No.: WHZXKYL2023-013). All participants provided written informed consent prior to enrollment. The study was conducted in accordance with the principles of the Declaration of Helsinki.

## 3 Results

### 3.1 DL-VMM system architecture and workflow

The deep learning-based vaginal microecological morphology assessment (DL-VMM) system comprises three sequential stages: data acquisition and preprocessing, model development, and component recognition with diagnostic output. A total of 500 clinical cases (from the enrolled patients) were used to construct a dataset containing approximately 12,000 annotated cells. High resolution images were acquired using a digital microscope scanner with a CMOS camera. Preprocessing included adaptive sharpening, normalization, and cell overlap segmentation, followed by region of interest extraction targeting epithelial areas with edge texture features. Ground truth labeling was performed by two experienced pathologists based on Amsel criteria and Nugent scoring. Data augmentation techniques, including Mixup, Cutout, color perturbation, and overlap simulation, were applied to enhance model robustness.

The core architecture is based on ConvNeXt-Base integrated with a spatial attention mechanism. A unified deep learning model was developed using multi-task joint learning to simultaneously perform detection, segmentation, and classification of key morphological components, with transfer learning applied using pre-trained weights from lineage cell datasets.

**FIGURE 1:**
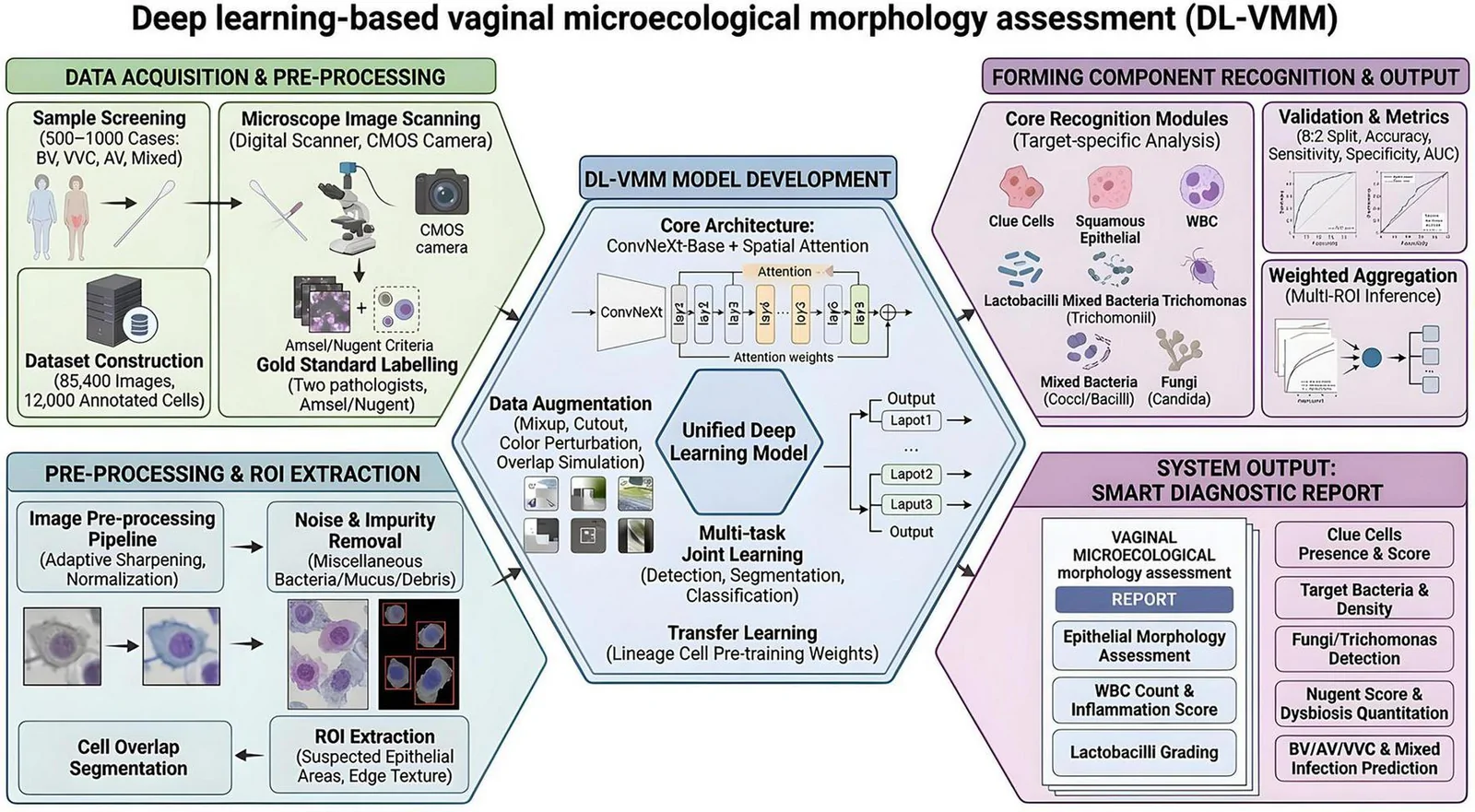
Workflow of the deep learning-based vaginal microecological morphology assessment (DL-VMM) system.

Target-specific recognition modules for clue cells and *Candida* fungi enable the system to generate a comprehensive smart diagnostic report, including vaginal microecological morphology assessment, epithelial morphology evaluation, white blood cell count with inflammation score, lactobacilli grading, clue cell presence and score, Nugent score with quantitative dysbiosis assessment for BV, target fungi detection, and VVC prediction. This integrated workflow facilitates automated, objective, and scalable diagnostic support for vaginitis evaluation (Figure 1).

### 3.2 Participant demographics and diagnostic comparison between routine wet mount microscopy and the GE6000 system

A total of 500 participants with vaginitis [all diagnosed with bacterial vaginosis (BV), vulvovaginal candidiasis (VVC), or mixed infection] were included. The mean age was 33.4 years (SD 6.8), and the median symptom duration was 4 days (IQR 2–7). Diagnostic performance of the GE6000 system was compared with routine wet mount microscopy. For BV, the GE6000 system detected 67 cases (13.4%) versus 62 cases (12.4%) by routine microscopy, showing no significant difference (*P* = 0.063). For VVC, detection rates were 149 (29.8%) with GE6000 and 146 (29.2%) with routine microscopy (*P* = 0.250). Mixed vaginitis (BV+VVC) was identified in 13 cases (2.6%) by GE6000 and 12 cases (2.4%) by routine microscopy (*P* = 0.564). Overall detection rates were 45.8% (229/500) for GE6000 and 44.0% (220/500) for routine microscopy. No statistically significant differences were observed between the two methods for any diagnostic category (all *P* > 0.05). These findings indicate that the GE6000 automated analyzer provides comparable diagnostic performance to routine wet mount microscopy for detecting BV, VVC, and mixed vaginitis in symptomatic patients (Table 2).

**TABLE 2:** Participant demographics and clinical characteristics (*n* = 500).

Characteristic	Value		
Age (years), mean (SD, standard deviation)	33.4 (6.8)		
Symptom duration (days), median (IQR, interquartile range)	4 (2–7)		
**Microbiological diagnosis, n (%)**	**Routine wet mount microscopy**	**GE6000**	***P*-value**
Bacterial vaginosis (BV)	62 (12.4%)*	67 (12.4%)*	0.063
Vulvovaginal candidiasis (VVC) *(based on Blastospores)*	146 (29.2%)*	149 (29.8%)*	0.250
Vulvovaginal candidiasis (VVC) *(based on Hyphae)*	163 (32.6%)*	167 (33.4%)*	0.125
Mixed vaginitis (BV + VVC)	12 (2.4%)*	13 (2.6%)*	0.564

SD, standard deviation; IQR, interquartile range. GE6000, automated vaginal secretion analyzer integrated with the DL-VMM system (#GE6000, Hunan Shengtong Medical Technology, China); DL-VMM, deep learning–based vaginal microecological morphology assessment.

*Percentages are based on the total number of participants (*N* = 500). Mixed vaginitis was defined as the concurrent presence of BV and VVC based on Gram stain and microscopic criteria.

### 3.3 Diagnostic performance of GE6000 (DL-VMM) and routine wet mount microscopy

For bacterial vaginosis diagnosed by clue cells, the GE6000 system achieved a sensitivity of 100.00% (95% CI: 94.19–100.00%), specificity of 98.86% (95% CI: 97.37–99.58%), and Kappa of 0.95 (95% CI: 0.91–0.99). For vulvovaginal candidiasis, using blastospores as the diagnostic criterion, GE6000 showed a sensitivity of 99.32% (95% CI: 95.85–99.96%), specificity of 98.87% (95% CI: 96.94–99.70%), and Kappa of 0.97 (95% CI: 0.94–0.99). When hyphae were used as the criterion, GE6000 demonstrated 100.00% sensitivity (95% CI: 97.68–100.00%) and 98.81% specificity (95% CI: 97.04–99.63%), with a Kappa of 0.98 (95% CI: 0.95–1.00) (Table 3). These findings collectively confirm the high diagnostic accuracy of the GE6000 system for both bacterial vaginosis and vulvovaginal candidiasis.

**TABLE 3:** Diagnostic performance of GE6000 (DL-VMM) versus routine wet mount microscopy for bacterial vaginosis and vulvovaginal candidiasis.

Condition	Method	Sensitivity (%)	Specificity (%)	AUC	PPV	NPV	Kappa
Bacterial vaginosis	DL-VMM	100.00 (94.19–100.00)	98.86 (97.37–99.58)	0.99 (0.98–1.00)	92.54 (83.97–96.90)	100.00 (98.81–100.00)	0.95 (0.91–0.99)
(based on clue cells)	Routine wet mount	100.00 (94.19–100.00)	100.00 (98.81–100.00)	1.00	100.00 (94.19–100.00)	100.00 (98.81–100.00)	1.00
Vulvovaginal candidiasis	DL-VMM	99.32 (95.85–99.96)	98.87 (96.94–99.70)	0.99 (0.98–1.00)	97.32 (92.95–99.04)	99.72 (98.24–99.97)	0.97 (0.94–0.99
*(based on Blastospores)*	Routine wet mount	100.00 (96.84–100.00)	100.00 (98.56–100.00)	1.00	100.00 (96.84–100.00)	100.00 (98.56–100.00)	1.00
Vulvovaginal candidiasis	DL-VMM	100.00 (97.68–100.00)	98.81 (97.04–99.63)	0.99 (0.98–1.00)	97.60 (93.78–99.14)	100.00 (98.54–100.00)	0.98 (0.95–1.00)
*(based on Hyphae)*	Routine wet mount	100.00 (97.68–100.00)	100.00 (98.54–100.00)	1.00	100.00 (97.68–100.00)	100.00 (98.54–100.00)	1.00

Data in parentheses are 95% confidence intervals. DL-VMM, deep learning–based vaginal microecological morphology assessment; AUC, area under the receiver operating characteristic curve; PPV, positive predictive value; NPV, negative predictive value.

### 3.4 Comparison of detection rates of morphological items between GE6000 and routine wet mount microscopy

**FIGURE 2:**
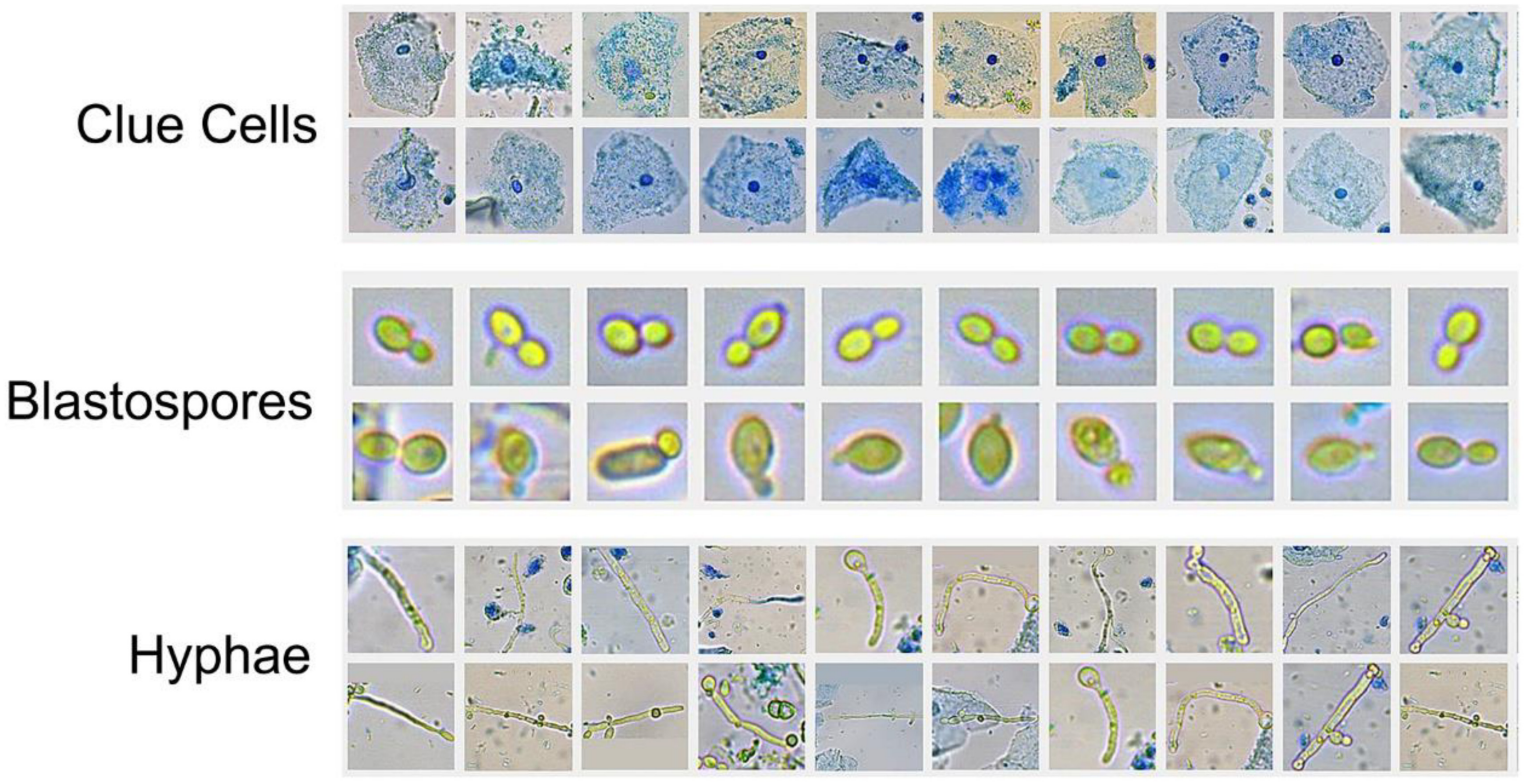
The representative morphologies of clue cells, blastospores and hyphae, as detected by the GE6000 system.

A total of 500 vaginal samples were analyzed to compare the detection rates of blastospores, hyphae, trichomonads, and clue cells between the GE6000 vaginal fluid analyzer equipped with the deep learning–based vaginal microecological morphology assessment system (DL-VMM, #GE6000, Hunan Shengtong Medical Technology, China) and routine wet mount microscopy. As summarized in Table 4, the detection rates were highly consistent between the two methods across all four parameters. For blastospores, manual microscopy identified 146 positive cases (29.20%), while GE6000 detected 149 positive cases (29.80%), showing no significant difference (χ^2^ = 0.043, *P* = 0.835). Hyphae were observed in 163 cases (32.60%) by manual microscopy and 167 cases (33.40%) by GE6000 (χ^2^ = 0.072, *P* = 0.788). Trichomonads were detected in 158 cases (31.60%) manually and 162 cases (32.40%) by GE6000 (χ^2^ = 0.074, *P* = 0.786). Clue cells were found in 62 cases (12.40%) by manual microscopy and 67 cases (13.40%) by GE6000 (χ^2^ = 0.223, *P* = 0.637). For all four parameters, the χ^2^ values were low, and all *P* values exceeded 0.05, indicating no statistically significant differences between the GE6000 system and routine wet mount microscopy. Figure 2 illustrates the representative morphologies of clue cells, blastospores, and hyphae as detected by the GE6000 system. These findings collectively demonstrate that the GE6000 automated analyzer achieves comparable detection performance to routine wet mount microscopy for key morphological parameters associated with vaginal infections.

**TABLE 4:** Comparison of detection rates of morphological items between fully automated vaginal secretion analyzer GE6000 and routine wet mount microscopy [n (%)].

Parameter	Manual microscopy		GE6000		χ ^2^-value	*P-*value
	Positive n (%)	Negative n (%)	Positive n (%)	Negative n (%)		
Blastospores	146 (29.20)	354 (70.80)	149 (29.80)	351 (70.20)	0.043	0.835
Hyphae	163 (32.60)	337 (67.40)	167 (33.40)	333 (66.60)	0.072	0.788
Trichomonads	158 (31.60)	342 (68.40)	162 (32.40)	338 (67.60)	0.074	0.786
Clue cells	62 (12.40)	438 (87.60)	67 (13.40)	433 (86.60)	0.223	0.637

GE6000, automated vaginal secretion analyzer integrated with the DL-VMM system (#GE6000, Hunan Shengtong Medical Technology, China); χ^2^, chi-square statistic (Pearson’s chi-square test, df = 1); *P*-value, from Pearson’s chi-square test comparing detection rates between GE6000 and manual microscopy.

### 3.5 Concordance between GE6000 and routine wet mount microscopy for seven morphological parameters

The concordance between the GE6000 deep learning–based vaginal microecological morphology assessment system and routine wet mount microscopy for seven morphological parameters is summarized in Table 5. The GE6000 system demonstrated substantial to almost perfect agreement across all parameters (according to Landis and Koch classification), with total agreement rates ranging from 96.40 to 99.60% and Kappa values between 0.89 and 0.98.

**TABLE 5:** Concordance between the GE6000 automated vaginal secretion analyzer integrated with the deep learning–based vaginal microecological morphology assessment (DL-VMM) system and routine wet mount microscopy for seven morphological parameters.

Parameter GE6000		Manual microscopy		Positive agreement rate (%)	Negative agreement rate (%)	Total agreement rate (%)	Kappa (95% CI)	AUC	Youden Index (%)	*P*-value
		Positive	Negative							
Epithelial cells	Positive	52	3	92.86 (52/56)	99.33 (441/444)	99.00 (493/498)	0.94 (0.90–0.98)	0.96	0.92	1.000
	Negative	4	441							
Leukocytes	Positive	75	2	97.40 (75/77)	100.00 (423/423)	99.60 (498/500)	0.98 (0.96–1.00)	0.99	0.97	0.999
	Negative	0	423							
Blastospores	Positive	145	4	99.32 (145/146)	98.87 (350/354)	99.00 (495/500)	0.97 (0.94–0.99)	0.99	0.98	0.349
	Negative	1	350							
Hyphae	Positive	163	4	100.00 (163/163)	98.81 (333/337)	99.20 (496/500)	0.98 (0.95–1.00)	0.99	0.99	0.504
	Negative	0	333							
Trichomonads	Positive	158	4	100.00 (158/158)	98.83 (338/342)	99.20 (496/500)	0.98 (0.95–1.00)	0.99	0.99	0.059
	Negative	0	338							
Clue cells	Positive	62	5	100.00 (62/62)	98.86 (433/438)	99.00 (495/500)	0.95 (0.91–0.99)	0.99	0.99	0.324
	Negative	0	433							
Cleanliness	Positive	380	8	97.44 (380/390)	92.73 (102/110)	96.40 (482/500)	0.89 (0.84–0.94)	0.95	0.90	1.000
	Negative	10	102							

GE6000, automated vaginal secretion analyzer integrated with the DL-VMM system (#GE6000, Hunan Shengtong Medical Technology, China); DL-VMM, deep learning–based vaginal microecological morphology assessment; AUC = area under the curve; Positive agreement rate = sensitivity; Negative agreement rate = specificity; Total agreement rate = accuracy; Youden Index = sensitivity + specificity – 1. Kappa values: < 0 = poor, 0.00–0.20 = slight, 0.21–0.40 = fair, 0.41–0.60 = moderate, 0.61–0.80 = substantial, 0.81–1.00 = almost perfect agreement. All *P*-values were calculated using the McNemar test or chi-square test.

For epithelial cells, total agreement was 99.00% (Kappa = 0.94). Leukocytes yielded 99.60% (Kappa = 0.98). Blastospores showed 99.00% (Kappa = 0.97), with sensitivity of 99.32% and specificity of 98.87%. Hyphae achieved 99.20% (Kappa = 0.98), with sensitivity of 100.00% and specificity of 98.81%. Trichomonads presented 99.20% (Kappa = 0.98). Clue cells showed 99.00% (Kappa = 0.95), with sensitivity of 100.00% and specificity of 98.86%. Cleanliness exhibited 96.40% (Kappa = 0.89), with sensitivity of 97.44% and specificity of 92.73%.

The AUC values ranged from 0.95 to 0.99, and Youden indices ranged from 0.90 to 0.99, further supporting the high diagnostic validity of the GE6000 system. *P*-values for all parameters were non-significant (range: 0.059 to 1.000), indicating no statistically significant difference between the two methods (McNemar test or chi-square test).

### 3.6 Analysis of discordant cases

To address the sources of disagreement, we examined the discordant pairs from Table 5. Overall, discordance was rare for most parameters. The highest discordance was observed for cleanliness grading, with 18 discordant cases out of 500 (3.6%): 10 were false-positive (DL-VMM positive, manual negative) and 8 were false-negative (DL-VMM negative, manual positive). For epithelial cells, there were 7 discordant cases (1.4%): 3 false-positive and 4 false-negative. For blastospores, 5 discordant cases (1.0%): 4 false-positive and 1 false-negative. For hyphae, 4 discordant cases (0.8%): all false-positive. For trichomonads, 4 discordant cases (0.8%): all false-positive. For clue cells, 5 discordant cases (1.0%): all false-positive. For leukocytes, only 2 discordant cases (0.4%): both false-positive.

These discordances did not cluster in any specific disease category or patient subgroup (e.g., age, symptom duration) based on exploratory analysis (data not shown). However, we noted that most false-positive DL-VMM calls for hyphae, trichomonads, and clue cells occurred in smears with abundant debris or overlapping cells, suggesting that morphological ambiguity may confound the algorithm. Conversely, the single false-negative for blastospores occurred in a sample with very low fungal burden (only a few yeast cells), indicating that low pathogen load remains a challenge for both automated and manual methods. Importantly, the inter-observer variability between the two human microscopists was not formally measured, but their consensus readings were used as reference; thus, some discordances could also reflect inherent subjectivity in manual interpretation. We provide a confusion matrix summary in Table 6 for all parameters.

**TABLE 6:** Confusion matrix summary for DL-VMM vs. manual microscopy (*N* = 500).

Parameter	TP	TN	FP	FN	Total discordant
Epithelial cells	52	441	3	4	7
Leukocytes	75	423	2	0	2
Blastospores	145	350	4	1	5
Hyphae	163	333	4	0	4
Trichomonads	158	338	4	0	4
Clue cells	62	433	5	0	5
Cleanliness	380	102	10	8	18

## 4 Discussion

In this prospective diagnostic study of 500 symptomatic women, we demonstrated that a deep learning–based vaginal microecological morphology assessment system (DL-VMM), integrated into the GE6000 automated analyzer, provides excellent diagnostic performance for bacterial vaginosis (BV) and vulvovaginal candidiasis (VVC). Notably, the system exhibited near-perfect concordance with routine wet mount microscopy across all key morphological parameters (Sobel, 1997; Workowski et al., 2021).

For BV diagnosed by clue cells, DL-VMM achieved a sensitivity of 100.00% (95% CI: 94.19–100.00%) and a specificity of 98.86% (95% CI: 97.37–99.58%), with a Cohen’s kappa of 0.95 (95% CI: 0.91–0.99). For VVC, depending on the diagnostic criterion (blastospores or hyphae), sensitivity ranged from 99.32 to 100.00%, specificity from 98.81 to 98.87%, and kappa values from 0.97 to 0.98. These metrics are comparable to, or exceed, those reported for other automated systems in reproductive infectious disease diagnostics (Lev-Sagie et al., 2023; Pongpom et al., 2026; Wang et al., 2021). The area under the curve (AUC) of 0.99 for both conditions further underscores the system’s high discriminative ability.

Beyond individual infection detection, DL-VMM consistently reproduced routine microscopy for seven morphological parameters: epithelial cells, leukocytes, blastospores, hyphae, trichomonads, clue cells, and cleanliness grade. Total agreement rates ranged from 96.40 to 99.60%, with kappa values between 0.89 and 0.98, indicating “almost perfect” agreement beyond chance. Notably, negative agreement rates (specificities) exceeded 98% for most parameters, except cleanliness (92.73%), suggesting that DL-VMM is particularly reliable for ruling out abnormalities. The nonsignificant results of the McNemar test (all *P* > 0.05) confirm that the two methods are statistically interchangeable for routine diagnostic purposes (Muzny and Kardas, 2020; Nugent et al., 1991).

However, we recognize that even a small discordance rate can be clinically meaningful. For example, the 3.6% discordance on cleanliness grading translates to 18 patients out of 500 who would receive different assessments of inflammation. Although many of these discrepancies involve borderline grades (II vs. III), they could influence decisions regarding antibiotic or anti-inflammatory therapy. Similarly, the 1.14% false negative rate for VVC (1 missed case among 149) and the 1.13% false positive rate for BV (5 false positive clue cell calls) are low but not negligible. Therefore, we recommend that in cases where clinical suspicion is high but DL-VMM returns a negative result, or when the automated report is equivocal (e.g., borderline cleanliness grade or ambiguous morphology), a manual confirmatory review should be performed. Our analysis of discordant cases suggests that most disagreements arose from overlapping cells, mucous debris, or low pathogen burden—conditions that also challenge human observers. Future algorithm improvements should focus on these challenging scenarios.

The clinical relevance of these findings is twofold. First, DL-VMM eliminates the operator dependence inherent to wet mount microscopy—a well-recognized limitation that reduces reproducibility, particularly in high-volume or low-resource settings (Amsel et al., 1983; Muzny and Kardas, 2020). Second, by providing automated quantitative outputs (e.g., dysbiosis indices and Nugent-compatible scores), DL-VMM supports standardized, microbiome-informed clinical decision-making. This represents a distinct advantage over binary or semiquantitative manual reads. As emphasized in prior research, understanding the broader vaginal microecological context—including lactobacilli density, clue cell abundance, and inflammatory markers—is essential for managing recurrent or mixed infections (Bradshaw et al., 2025; De Seta et al., 2025). DL-VMM directly addresses this gap.

Several limitations should be acknowledged. First, the study was conducted at a single center in China, which may limit generalizability to populations with different vaginal microbiota profiles (Bradshaw et al., 2025). Multi-center external validation is needed to confirm the robustness of the model across diverse ethnic and geographic groups. Second, the reference standard incorporated manual microscopy-dependent components (Nugent score, fungal microscopy), potentially introducing incorporation bias. However, the use of a composite reference standard (combining clinical and microbiological criteria) partly mitigates this concern (Amsel et al., 1983; Nugent et al., 1991). Third, while DL-VMM demonstrated excellent performance for BV and VVC, its utility for other conditions—such as aerobic vaginitis or low-burden trichomoniasis—requires further validation (Lev-Sagie et al., 2023; Workowski et al., 2021). Fourth, the economic and workflow integration aspects of deploying DL-VMM in real-world laboratory settings were not assessed in this study. Fifth, inter-observer variability between human microscopists was not quantified; our consensus-based reference may not fully capture the inherent subjectivity of manual reading, and some discordances between DL-VMM and the consensus could reflect disagreement that would have occurred between individual microscopists.

Despite these limitations, our results indicate that DL-VMM is a robust, accurate, and objective alternative to routine wet mount microscopy for the diagnosis of BV and VVC. The system’s ability to generate a comprehensive microecological report within seconds could facilitate same-day, standardized vaginitis management. Future research should focus on external validation in diverse populations, cost-effectiveness analyses, and integration with antimicrobial susceptibility testing or microbiome sequencing data (Bradshaw et al., 2025; De Seta et al., 2025; Pongpom et al., 2026).

## 5 Conclusion

This prospective diagnostic study demonstrates that the deep learning–based vaginal microecological morphology assessment system (DL-VMM, GE6000) achieves excellent diagnostic accuracy for bacterial vaginosis and vulvovaginal candidiasis, with near-perfect agreement with routine wet mount microscopy. By eliminating operator dependence, providing rapid and objective results, and offering a comprehensive assessment of vaginal microecology, the automated system has the potential to standardize vaginitis diagnosis and improve clinical decision-making in routine practice. *Clinicians should, however, be aware of the small but clinically relevant discordance rates, particularly for cleanliness grading, and consider manual review for borderline or unexpected results.*

## Data Availability

The raw data supporting the conclusions of this article will be made available by the authors, without undue reservation.
